# Deep Learning Based Prediction of Pulmonary Hypertension in Newborns Using Echocardiograms

**DOI:** 10.1007/s11263-024-01996-x

**Published:** 2024-02-06

**Authors:** Hanna Ragnarsdottir, Ece Ozkan, Holger Michel, Kieran Chin-Cheong, Laura Manduchi, Sven Wellmann, Julia E. Vogt

**Affiliations:** 1 Department of Computer Science, ETH Zurich, Universitätstrasse 6, 8092 Zürich, Switzerland; 2 Department of Brain and Cognitive Sciences, Massachusetts Institute of Technology, 43 Vassar Street, Cambridge, MA 02139 USA; 3 Department of Neonatology, University Children’s Hospital Regensburg (KUNO), Hospital St. Hedwig of the Order of St. John, University of Regensburg, Regensburg, Germany

**Keywords:** Echocardiography, Computer assisted diagnosis, Explainable machine learning, Pulmonary hypertension, Pediatrics

## Abstract

Pulmonary hypertension (PH) in newborns and infants is a complex condition associated with several pulmonary, cardiac, and systemic diseases contributing to morbidity and mortality. Thus, accurate and early detection of PH and the classification of its severity is crucial for appropriate and successful management. Using echocardiography, the primary diagnostic tool in pediatrics, human assessment is both time-consuming and expertise-demanding, raising the need for an automated approach. Little effort has been directed towards automatic assessment of PH using echocardiography, and the few proposed methods only focus on binary PH classification on the adult population. In this work, we present an explainable multi-view video-based deep learning approach to predict and classify the severity of PH for a cohort of 270 newborns using echocardiograms. We use spatio-temporal convolutional architectures for the prediction of PH from each view, and aggregate the predictions of the different views using majority voting. Our results show a mean F1-score of 0.84 for severity prediction and 0.92 for binary detection using 10-fold cross-validation and 0.63 for severity prediction and 0.78 for binary detection on the held-out test set. We complement our predictions with saliency maps and show that the learned model focuses on clinically relevant cardiac structures, motivating its usage in clinical practice. To the best of our knowledge, this is the first work for an automated assessment of PH in newborns using echocardiograms.

## Introduction

Pulmonary hypertension (PH) is a rare but complex and progressive condition of the pulmonary arterioles that can affect newborns and children as well as adults. Functional and anatomical changes increase pulmonary artery pressure (PAP) and PH is formally defined as an increased mean PAP at rest with mPAP $> 20\,\hbox {mm}\,\hbox {Hg}$ (Simonneau et al., 2019). The level of PAP in newborns is frequently high and is expected to decrease after birth to reach a level comparable to healthy adult values (de Boode et al., 2018). When normal cardiopulmonary transition fails to occur, the newborns are affected by persistent pulmonary hypertension of the newborn (PPHN). PH is associated with bronchopulmonary dysplasia in older premature infants and various chronic pulmonary, cardiac, and systemic diseases for newborns at term contributing to morbidity and mortality (EL-Khuffash, 2014; Hansmann, 2017; Steinhorn, 2010). The PH prognosis is associated with the severity of the disease at diagnosis; thus, delayed treatment decreases the chance of survival (Barst et al., 2012; Żuk et al., 2016).

The gold standard for PH diagnosis is right heart catheterisation (RHC). However, due to the invasive nature of this costly procedure and the resulting high risk of related complications, especially in the pediatric age group (Rosenkranz & Preston, 2015), RHC is not a screening procedure. Transthoracic echocardiography performed by experts is instead the recommended non-invasive diagnostic tool for estimating the likelihood of PH and the severity of PH (Ni et al., 2019). This technique involves capturing a sequence of ultrasound images of the beating heart from different angles of the heart (views), obtaining by placing the transducer in different locations. It is one of the most common and growing diagnostic tools due to its low-cost, portable, and non-invasive technology, which makes it an ideal choice for pediatrics (Lang et al., 2015).

Different echocardiography modes are available, including 2D, 3D and Doppler. Although 3D echocardiography can give more information about the assessed region and is superior to 2D echocardiography, it has yet to translate to routine clinical usage (Hur & Sugeng, 2019). Thus, PH evaluation is commonly done using either 2D echocardiography videos (ECHOs) or Doppler echocardiograms (Lang et al., 2015; Ni et al., 2019). Using Doppler, screening of PH typically involves estimating PAP; however, the measurements may frequently be inaccurate. Thus, it is not the ultimate predictive tool to assess and manage PH (Fisher et al., 2009). Since elevated PAP can result in abnormalities in the shape and structure of the heart, subjective evaluation is often performed to detect the changes using ECHOs (Galiè et al., 2015).

Human assessment of PH using echocardiography with the procedures mentioned above is both time-consuming and expertise-demanding, which may delay care to a more advanced stage of illness, potentially decreasing the chance of survival (Barst et al., 2012). Thus, there is a clear need for an automatic and streamlined method to assist clinicians in assessing PH in newborns. Currently, little effort is directed toward automatic approaches for PH diagnostics; thus, this demand still needs to be met. The few existing methods from the literature for automatic PH prediction are only proposed for the adult population and do not assess the PH severity nor explain their predictions (Zhang et al., 2018; Leha et al., 2019).

In this work, we are interested in not only having an automated method to predict the existence of PH but also to classify its severity, as the severity plays a key role in the appropriate PH treatment strategy (Dasgupta et al., 2021; Fisher et al., 2009). We aim at exploring the importance of the various factors contributing to the solution, including the effects of known deep learning techniques, such as data augmentation and regularisation and the effects of various domain-specific factors. These include the exploration of the effect of including ECHOs from different views of the heart and the effect of temporal component of the ECHO videos. Finally, we seek to explain the predictions regarding cardiac structures and features, both to increase our understanding of the method and to increase the trust in the automatic prediction, ensuring clinical usability.

Accordingly, we propose a robust and explainable deep learning approach to predict and classify the severity of PH by utilising spatio-temporal patterns of the ECHOs from multiple views. To the best of our knowledge, this is the first work on multi-view video-based automated assessment of PH in newborns. To increase its clinical usability, we complement our predictions with saliency maps highlighting how the learned model focuses on clinically relevant cardiac structures. We show that these learned localization maps align with how clinicians subjectively assess PH.

To ensure the reproducibility of our work, the code was made publicly available under https://github.com/hanna15/echo_classification.

## Background

PH assessment is commonly performed manually on 2D or Doppler ECHO by measuring echocardiographic variables and/or by subjective ECHO evaluation. We will discuss the quantitative and the qualitative approaches for human assessment of PH in more detail below. Afterwards, we will walk the reader through the few existing Machine Learning (ML) approaches for PH prediction. We will conclude the section with a brief discussion on the importance of explainable ML for healthcare.

### Human Assessment of PH in Newborns

To assess the heart’s condition, a clinician records an echocardiogram containing a sequence of ultrasound images of the patient’s heart at a specific view. The three major views include a parasternal long-axis view (PLAX), an apical four chamber view (A4C), and a parasternal short-axis view at the level of papillary muscles (PSAX-P).

**Fig. 1:**
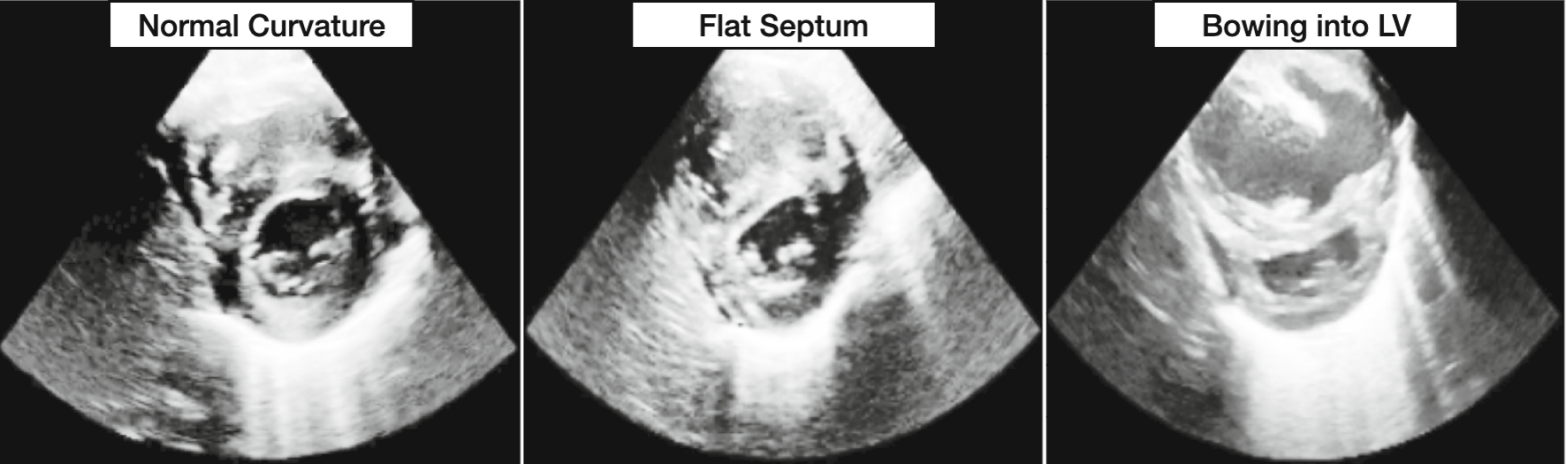
Varying septal morphology depending on the degree of PH on the PSAX-P view during systole (minimum expansion of the heart). Left: No PH, Middle: Mild PH, Right: Severe PH. The examples are taken from our dataset

#### Quantitative Evaluation

PH estimation in newborns frequently involves measuring various echocardiographic variables of 2D and Doppler echocardiography that allow for estimating mPAP. The traditional approach assumes the presence of tricuspid regurgitation (TR) in PH patients and relies on measuring the TR velocity (TRV) from Doppler of A4C and/or PLAX views. Systolic PAP (sPAP) can then be estimated from the TRV and right atrial pressure (RAP), as described in Eq. (1). Importantly, RAP is not measured but estimated from inferior vena cava (IVC) diameter and inspiratory collapse. Finally, mPAP has a strong linear relationship with sPAP, and can be derived as Brugger et al. (2021):

1 $$\begin{aligned} {\textit{sPAP}}= & {} 4*({\textit{TRV}})^2 + {\textit{RAP}}\end{aligned}$$

2 $$\begin{aligned} {\textit{mPAP}}= & {} 0.61*{\textit{sPAP}} + 2\,\mathrm{mm\,Hg} \end{aligned}$$

Previous studies have demonstrated that the agreement between PAP estimated from TRV and invasively measured PAP is only moderate (D’Alto et al., 2013; Fisher et al., 2009; Greiner et al., 2014). At an individual level, there can be significant under- and overestimation, potentially leading to misdiagnosis and inappropriate treatment (Augustine et al., 2018). This can happen due to many factors: (i) as TRV is squared in Eq. (1) even small errors in the absolute measurement of TRV can result in significant changes to the estimate of sPAP, (ii) in many patients, IVC dimensions for RAP estimation cannot be obtained, (iii) absence of TR is insufficient to exclude the presence of PH. For example, a recent study has shown that for patients that are both referred for RHC and ECHO, PH (as determined by RHC) is present in nearly half of the patients without a measurable TR velocity (O’Leary et al., 2018). Measurements of further variables are thus recommended, especially in the absence of measurable TRV. These include variables measured from 2D ECHOs, such as the left atrial (LA) to aortic ratio (LA:Ao) from the PLAX view, which correlates with increased pulmonary flow (EL-Khuffash, 2014). However, given that no single variable has been detected as the definitive predictive parameter to assess PH, and because the measurement may be frequently inaccurate, quantitative evaluation of 2D or Doppler ECHO is not the ultimate predictive tool, despite its widespread use (Fisher et al., 2009).

#### Qualitative Evaluation

Since elevated PAP can result in abnormalities in the shape and structure of the heart, visual and subjective evaluation on ECHOs is also commonly performed for estimating PH (EL-Khuffash, 2014; Galiè et al., 2015). The parasternal short axis view (PSAX) is specifically suitable for a subjective echocardiography evaluation of PH in newborns. From this view, abnormalities can, for example, be detected in the shape of the interventricular septum (IVS) and left ventricle during minimum expansion of the heart (systole). In a normal heart, the IVS is round, but becomes flat in patients with mild PH, and in severe PH the left-ventricle becomes D-shaped, or crescentic, as seen in Fig. 1.

During maximum expansion (diastole), reversed volume of the ventricles can also be detected (EL-Khuffash, 2014). Other views, such as the parasternal long axis view (PLAX) and the apical four-chamber view (A4C), can also be utilized for subjective evaluation of PH in newborns. Changes in right-ventricular size and hypertrophia of the right ventricular wall can be seen from the PLAX view, and from the A4C view changes in the right-ventricular area are often detected in case of moderate and severe PH (EL-Khuffash, 2014).

### Automated PH Predictions

Several ML methods have been proposed to automatically estimate PH in adults using different input modalities, such as chest X-rays (Kusunose et al., 2020; Zou et al., 2020), ECGs (Kwon et al., 2020; Mori et al., 2021; Aras et al., 2023), heart sounds recorded by acoustic sensors (Kaddoura et al., 2016), CTs (Sharkey et al., 2022; Vainio et al., 2021), EHRs (Kogan et al., 2023), and MRIs (Bello et al., 2019; Dawes et al., 2017). Even though echocardiography is the recommended non-invasive modality for PH estimation, and the most common routine test used in newborns to diagnose or rule out various heart diseases (Ni et al., 2019), not much effort has been directed towards the automatic assessment of PH using echocardiography. The exceptions are the work of Leha et al. (2019), Diller et al. (2022), and Zhang et al. (2018), which propose methods for automatic PH prediction in the adult population.

The authors in Leha et al. (2019) propose an approach that relies on manually extracted ECHO parameters and applies various ML algorithms, such as regression and SVM in order to predict PH. The goal of their method is to help standardize and simplify integration of the several parameters that relate to PH. The main drawback is that the ECHO parameters must still be measured and estimated by highly trained specialists limiting its applicability in a real-world scenario.

The work of Zhang et al. (2018) shows the potential of using deep learning for predicting PH using ECHOs, requiring no manual feature extraction. This method, however, has several limitations. First of all, it only uses a single A4C view of the heart, although the literature has shown that considering multiple views improves accuracy for the manual prediction of PH (Schneider et al., 2017). Second, it works on static frames of the ECHO videos and does not exploit the spatio-temporal patterns in the ECHO sequence, although spatio-temporal deep learning methods have shown superior results for various video classification tasks. Similarly, Diller et al. (2022) use deep learning to estimate PH based on static ECHO frames. Specifically, they propose to train a model to segment cardiac chambers and extract geometric information throughout the cardiac cycle. The ensemble of deep convolutional networks estimates RV systolic pressure (RVSP), as a predictor of PH. Finally, similar to the existing approaches for PH prediction from other modalities, Zhang et al. (2018) has limited accountability and clinical usability. The reason is twofold; first, the black-box nature of these approaches makes their internal mechanisms and their results opaque, and second, they focus only on binary PH classification but do not predict the PH severity. Severity estimation of PH is of great clinical importance, as guidelines for PH treatment type and urgency depend on PH severity (Corris & Degano, 2014; Galiè et al., 2015).

### Explainable ML in Healthcare

In recent years, interpretability and explainability of machine learning (ML) models have attracted much attention. Various methods have been proposed to help explain the reasons for a model’s prediction, which is vital for applying ML to healthcare, where achieving high predictive accuracy is often as important as understanding the prediction. Indeed, the lack of explainability is a critical factor that limits the wider adoption of ML in healthcare, as without it, medical practitioners often find it challenging to trust ML models (Stiglic et al., 2020).

Although explainability and interpretability of ML models are often used interchangeably, interpretability technically refers to the extent to which a human can understand a model independently. In contrast, explainability refers to the extent to which the internal mechanics of a model can be (post-hoc) explained in human terms—usually for models which are too complicated to be understood by humans (Rudin, 2019). In this work, we will be focusing on the explainability of the methods to explain the convolutional neural networks (CNNs), one of the most common deep learning methods for medical image understanding. Their black-box nature, however, limits clinical usability.

**Table 1:** Characteristics of the dataset. It includes 192 newborns and 936 2D ECHOs for train and validation and 78 newborns and 375 ECHOs for the test set from 5 different standard views

Data split	Feature	Value
Train + Validation	PH (#None (%)/#Mild (%)/#Severe (%))	126 (65%)/32 (17%)/34 (18%)
	Age (days) ($\text {Mean} \pm \text {SD}$)	$56 \pm 160$
	Maturity in birth (days) ($\text {Mean} \pm \text {SD}$)	$230 \pm 46$
	Patient’s weight (kg) ($\text {Mean} \pm \text {SD}$)	$2.9 \pm 1.5$
Test	PH (#None (%)/#Mild (%)/#Severe (%))	55 (70%)/9 (12%)/14 (18%)
	Age (days) ($\text {Mean} \pm \text {SD}$)	$34 \pm 42$
	Maturity in birth (days) ($\text {Mean} \pm \text {SD}$)	$206 \pm 32$
	Patient’s weight (kg) ($\text {Mean} \pm \text {SD}$)	$2.1 \pm 1.2$
Train + Validation + Test	Spatial size of original 2D images (pixels)	1440 x 866
	Video length (frames) ($\text {Mean} \pm \text {SD}$)	$122 \pm 2$
	Video FPS	25 fps
	Manufacturer (ultrasound machine/transducer)	GE Logic S8/S4–10 at 6 MHz

Recently, several works have proposed explainability of CNNs by using *visual explanation methods*, which identify and visualize the contribution of each pixel to the output of the trained network (Molnar, 2022). Generally, the results are expressed as an importance map (often referred to as salience or attribution map) of the same size as the input image, where each scalar in the map quantifies the contribution of the corresponding pixel (Li et al., 2021; Molnar, 2022). The explanations are either generated by perturbing parts of the image and observing the change of the prediction (*perturbation-based methods*) or by computing the gradient of the prediction with respect to input features (*gradient-based methods*). The gradient-based methods are commonly used, since they are faster to compute. However, the perturbation-based methods have the benefit of not requiring access to the intermediate layers (Molnar, 2022). A vast number of gradient-based methods have been proposed, including Vanilla Gradients (Simonyan et al., 2014), DeconvNet (Zeiler & Fergus, 2014), and Grad-CAM (Selvaraju et al., 2017). While they have been shown to explain model decisions (Selvaraju et al., 2017; Lanfredi et al., 2021), some of those methods have also been shown to be insensitive to models and data, acting more like edge detectors by simply highlighting strong pixel changes in images. Of the tested explainability methods in Adebayo et al. (2018), Ghorbani et al. (2019), only Vanilla Gradients and Grad-CAM passed the insensitivity check, making them the preferred methods.

In medical imaging, the predictions of CNNs can be further explained by utilising expert- and domain-specific medical knowledge (Zhu & Ogino, 2019; Lee et al., 2019). The first application of interpretation frameworks to understand deep learning models from ECHOs has just recently been proposed (Ghorbani et al., 2020). Using visual explanation methods, they show that their models (trained on static ECHO frames) pay appropriate attention to key cardiac structures when performing human-explainable tasks, such as detecting the presence of pacemaker and defibrillator leads. However, no effort has yet been made to explain the predictions of models assessing PH, and no published work has shown spatio-temporal explanation of ECHO sequences. Indeed, most work on explainable ML is centered around spatial input. However, visual explanation approaches have recently been shown to be expandable to 3D-CNNs trained on video clips (Li et al., 2021). For example, Stergiou et al. (2019a, 2019b) recently adapted Class Activation Mapping (CAM) for 3D-CNNs, such that important spatio-temporal regions of the input videos are highlighted.

## Methods

In this section, we first introduce the dataset employed in this work, along with the preprocessing and data augmentation steps undertaken. We then discuss the spatial approach and introduce our proposed spatio-temporal approach in both single- and multi-view settings. Finally, this section will conclude with the explainability method adapted in this work.

### Dataset

The dataset in this work was collected in two batches. The first batch consists of 936 2D transthoracic echocardiography videos (ECHOs) from 192 newborns, taken from a single center (the Hospital Barmherzige Brüder Regensburg), between the years 2019–2020 and used as the training and validation set. The second batch was collected in 2022 at the same center, has 375 ECHOs and serves as the held-out test set with 78 newborns. The ECHOs were performed by a senior pediatric cardiologist using a GE Logic S8 ultrasound machine with the S4–10 transducer at 6 MHz frequency.

Each ECHO consists of a sequence of ultrasound images of the patient’s heart from one of five standard views: PLAX, A4C, and three parasternal short-axis views; at the level of papillary muscles (PSAX-P), at the level of semilunar valves (PSAX-S), and on the apical short-axis view (PSAX-A). The ECHO videos are recorded at 25 frames per second with an average length of 5 s, covering approximately ten heartbeats. The spatial resolution of the videos is $1440\times 866$.

The ground truth annotations for each ECHO were determined through visual evaluation by a senior pediatric cardiologist. The ground truth labels differentiate between three levels of pulmonary hypertension (PH). For the first batch the levels are none (65%), mild (17%), and moderate to severe (18%) PH, and for the second batch none (70%), mild (12%), and moderate to severe (18%) PH. Grading was done using PSAX-P view, annotating no PH if there is no septal flattening; mild PH, if there is a decent septal flattening (curvature into the right ventricle); and moderate to severe PH if the septum is bowing into the left ventricle, as visualised in Fig. 1. Furthermore, for each ECHO, its corresponding view is also annotated. Note that the dataset is inherently imbalanced as mild and severe cases are rare compared to healthy cases, increasing the complexity of the problem.

A detailed overview of the data for both of the batches is provided in Table 1. The conduct of this study was approved by the local ethics committee and all collected data was pseudonymized to protect the privacy.

### Preprocessing and Data Augmentation

As the first step in preprocessing the available data, we crop and mask the ECHOs to eliminate any extraneous information (additional text or signals) outside the scanning sector and resize them to $224\times 224$ pixels using bilinear interpolation. A histogram equalization technique is then applied to distribute the pixel intensities across the full range of gray-scale values and normalize them.

During training, various image transformations are applied to improve the robustness of the learned model. The first type of transformation is intensity transformations, designed to make the model invariant to variations in intensity or brightness levels in the ECHOs. The second type of transformation is spatial transformations, which are applied to increase resilience against different zoom settings of the ultrasound machine, variations in the actual size of the heart being imaged, and different transducer placements.

In particular, we apply sharpness and brightness adjustments, Gamma correction, noise addition and background variation as intensity transformations and rotation, translation and re-scaling as spatial transformations. These random transformations are applied to each sequence, where each transformation has a probability of 0.5:

1. *Sharpness adjustments:* Sharpening the image by up to 8x, or blurring it with a sharpness factor $(f)<1.0$, specifically $f \in [0,0.9999]$.
2. *Brightness adjustments:* Using an enhancement factor between 0.5 and 1.2 (a factor $<1$ gives a darker image, a factor $>1.0$ a brighter image and 1.0 the original image).
3. *Gamma correction:* Using a gamma factor ($\gamma$) between 0.25 and 2.0, where the gamma correction ($I_{\gamma }$) of an input image $I_{in}$ is given by: $I_{\gamma } = 255 * (I_{in}/255)^\gamma$
4. *Addition of noise:* Salt and pepper noise, with a threshold of 0.005, and random Gaussian noise.
5. *Background variation:* Using different amounts of speckle noise.
6. *Rotation and translation:* Rotate between $-15^\circ$ to $15^\circ$, and translate up to 0.1$\times$.
7. *Re-scaling:* Scaling down to 0.8$\times$, and zooming up to 1.2$\times$.

**Fig. 2:**
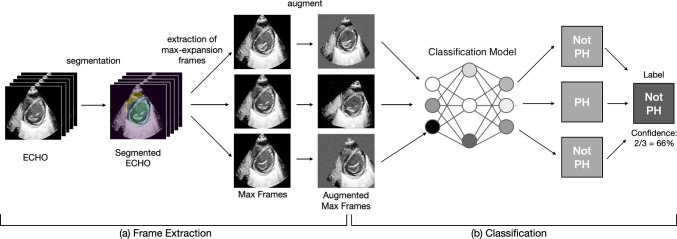
Overview of the spatial method for PH detection using a single view (PSAXP-P), when training on e.g. three maximum-expansion frames per ECHO (shown only for a single patient for simplicity). The first step involves **a** extracting maximum-expansion frames using segmentation models. The next step involves **b** classification using the extracted frames

In this work, the dataset is very imbalanced, a very common challenge in machine learning applications, especially in healthcare. Strategies to address this imbalance have been extensively studied, and one commonly used approach involves sampling methods (Buda et al., 2018). To address this issue, we apply random resampling with replacement, in combination with the random augmentations described above, during training. More specifically, we process approximately an equal number of samples from each class for each epoch, ensuring the model receives a balanced representation of the samples from all classes. To achieve this, we employ a weighted random sampler sampling elements from the dataset with replacement from indices $i = [0,\ldots , N]$, with probabilities given by the weights $W = [w_0, \ldots , w_N]$, using weight $w_i$ of each sample. Note that the oversampling procedure will not produce identical copies of the same samples because the data augmentation slightly modifies each sample.

### Single-View Spatial Approach

We first implement a spatial-only model using a single-view ECHO for PH prediction for newborns by training a convolutional neural network on manually curated frames from ECHO videos. To overcome the scarcity of the annotated data, we extract *n* frames from each ECHO, using different frame extraction heuristics, as further explained in Sect. 3.3.1. Thus, each frame is considered an individual sample for the classification, giving rise to frame-level predictions, whose results are aggregated to achieve view-level predictions, as explained in Sect. 3.3.2. The training details and the model architecture are further described in the following sections. An overview of the spatial approach is provided in Fig. 2, both for the (a) frame extraction phase and (b) classification phase.

#### Frame Extraction

We propose three different methods to extract training frames from each ECHO. As a first approach, we select *n* frames at random. As a second and third approach, we use domain knowledge to select the most representative frames that are most relevant for assessing PH by cardiologists, specifically those corresponding to systole (minimum expansion) where variations in septal morphology due to PH are most prominent, but potentially also those corresponding to diastole (heart’s maximum expansion) (EL-Khuffash, 2014). We explore two methods for identifying these frames: an algorithmic approach based on a cardiac phase detection algorithm (Zhang et al., 2017) with parameters optimized for newborns and a neural network-based image segmentation approach. However, the algorithmic approach proved imprecise due to small errors in heart-phase estimation in the first cycle accumulating in subsequent cycles. Thus, we moved forward with the neural network-based image segmentation method.

More concretely, we identify the pixels corresponding to the left ventricle (LV) and right ventricle (RV) using the output of the segmentation model. We then calculate the relative area of the ventricles in each frame by dividing the number of pixels corresponding to the ventricles by the total number of pixels. The minimum and maximum expansion frames were then identified with the smallest or largest relative ventricle area. For getting the segmentation masks, we apply ECHO segmentation models from Zhang et al. (2018), which were trained on adult ECHOs in five standard views, including PSAX-P, PLAX, and A4. The intersection over union (IoU) scores for the views and segmentation areas of interest on the local test data from Zhang et al. (2018) ranged from 64.6 to 88.9. However, these models were trained on a dataset with a mean subject age of 59 (Zhang et al., 2018). In contrast, our dataset population consists of newborns; thus, lower performance is to be expected on our dataset. Due to the lack of segmentation ground truth for our data, this assumption cannot be verified numerically. However, visual inspection approves this. Since we are only interested in the joint area of the LV and RV relative to the rest of the heart, perfect segmentation of each ventricle is not necessary.

#### Spatial Classification

For the PH classification on spatial input, we train a convolutional neural network with residual connections on *n* frames per ECHO, yielding a prediction for each frame. To get view-level results, we aggregate frame-level predictions of a given view $\{y_{\text {view}, i}\}_{i=1,\ldots , n}$ through majority voting, i.e. by selecting the most frequently predicted label as the view-level prediction $y_{\text {view}}$. The view-level confidence is then defined as $C = y^{*}_{\text {view}} / n$, where $y^{*}_{\text {view}}$ is the count of the most frequently predicted label from the list of predictions for the *n* frames of a given ECHO per view. Figure 2b summarises the classification process when training on maximum-expansion frames after the maximum-expansion frames have been extracted and augmented. Note that the process is the same for training on minimum-expansion frames, but for training on random frames, the segmentation step can be excluded.

### Proposed Spatio-Temporal Approach

**Fig. 3:**
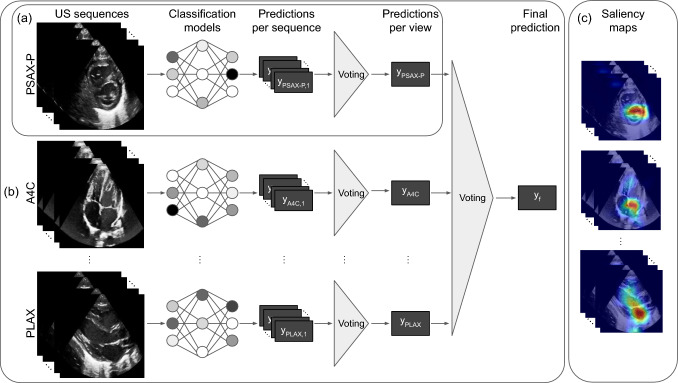
Overview of our proposed method to automatically assess PH severity of a patient using **a** single view and **b** multi-view approach with majority voting utilising spatio-temporal patterns of ECHOs. Spatio-temporal saliency maps **c** are provided from each view to increase clinical usability

In contrast to previous work Zhang et al. (2018) using a single-view temporal approach, we introduce a spatio-temporal multi-view end-to-end deep learning model. We assume that, we have access to multiple ECHOs showing the heart from different views for each patient, which should contribute to the patient-level prediction. Even though a single view is commonly used for the subjective assessment, recent works have shown that using multiple views is beneficial for the assessment (EL-Khuffash, 2014; Schneider et al., 2017). The proposed framework is depicted in Fig. 3.

#### Single-View

We first process each view separately. In particular, we employ a 3D-CNN architecture with residual connections and spatio-temporal convolutions across frames Hara et al. (2017) (see Fig. 3a). In contrast to previous work (Zhang et al., 2018), our approach integrates spatial as well as temporal information into the learning process. This mitigates the frame-level variations that can occur due to external changes, such as the position or the contact of the transducer or the cardiac function itself, thereby increasing robustness. To overcome the scarcity of the annotated data, common in the medical domain, from each ECHO, we extract *n* shorter video sequences by randomly choosing a frame as their starting frame followed by $k-1$ consecutive frames (using a sampling interval of $s=1$), with total *k* frames, covering on average one heartbeat. Sequence-level predictions $\{y_{\text {view}, i}\}_{i=1,\ldots , n}$ are then aggregated through majority voting, i.e., by selecting the most frequently predicted label, to a view-level prediction $y_{\text {view}}$.

The view-level confidence is then defined in a similar manner as for the spatial approach, that is: $C = y^{*}_{\text {view}} / n$, where $y^{*}_{\text {view}}$ is the count of the most frequently predicted label from the list of predictions for the *n* sequences of a given ECHO per view.

#### Multi-view

To increase the robustness of the method further, we employ a multi-view approach by combining the models trained on each available view (as in Fig. 3b). The final subject-level prediction, $y_{\text {f}}$, is then achieved by majority voting of the view-level predictions. In the case of a tie, the prediction of the model(s) with higher confidence is selected. We tried different approaches for view aggregation, including feature-level fusion, where modalities are combined in the embedding space. This is done by learning intermediate features for each view and these are then combined and jointly modeled to make a decision. However, the feature-level fusion is memory heavy and did not provide improvements compared to simple majority voting.

Note that our proposed method uses view annotations to differentiate the distinct modalities. Following recent work on view classification (Zhang et al., 2018), our method can easily be extended to incorporate ECHOs without view annotation. Furthermore, both spatial and spatio-temporal can be used with multi-view setting.

#### Explainability

To increase the accountability and clinical usability of our proposed method, we complement our predictions with spatio-temporal saliency maps from each view (as in Fig. 3c). The automatic localisation of relevant pixels in the video sequence for the model’s prediction provides explanations that mimic the clinical workflow. Among different methods (Selvaraju et al., 2017; Springenberg et al., 2015; Zhou et al., 2016), we chose to use Grad-CAM (Gradient-weighted Class Activation Mapping). This method exploits the gradients of target concept flowing into a given convolutional layer to produce a coarse localization map highlighting the important regions in the image for predicting the label Selvaraju et al. (2017). Recent works Kindermans et al. (2019), Adebayo et al. (2018) have shown how pixel-space gradient visualizations, such as Guided Backpropagation and Guided Grad-CAM, could be rather insensitive to model and data, making them similar to edge detectors. Grad-CAM is one of few saliency methods that pass the insensitivity check, making it our saliency method of choice (Adebayo et al., 2018).

Note that Grad-CAM was originally proposed for 2D-CNNs. We extend Grad-CAM to 3D-CNNs processing spatio-temporal video inputs. This allows us to identify the spatio-temporal regions on the video sequence that the network finds most informative for its prediction, which are the regions in spatial and time domains. We do this by assigning each neuron a relevance score for the class prediction at the output layer. Then, we backpropagate this information to the last convolutional layer to produce a coarse spatio-temporal localization map highlighting spatio-temporal areas of the ECHO video sequence.

## Experiments and Results

### Implementation Details

Our method was developed using the Python programming language with the PyTorch deep learning library. Experiments were run on a cluster containing different NVIDIA GeForce graphic cards: GTX 1080, GTX 1080 Ti, RTX 2080 Ti with 2048 MB RAM per processor core.

For both spatial and spatio-temporal approach we used a ResNet-18 as the model backbone (Carreira & Zisserman, 2017), and initialise the model weights with pre-trained weights from ImageNet (Russakovsky et al., 2014). For the spatio-temporal approach we extracted $n=10$ video sequences for each view, and each sequence was composed of $k=12$ consecutive frames, covering on average one heartbeat in every sequence. The sequence length, *k*, and sampling interval *s* were determined empirically (see Sect. 4.4.2). For the spatial approach, we extracted $n=10$ frames, with different extraction method, see Sect. 4.3. To deal with class imbalance, we employed a weighted random sampler, which samples elements from the dataset using their inverse class weight as their sample weight, ensuring that for every epoch, the model sees approximately equal number of samples from each class. Additionally, during training, we continuously augmented each sample with a probability of 90%, as selected empirically. Each model was trained for around 150 epochs per view minimizing the (categorical) cross-entropy loss with the Adam (Kingma & Ba, 2014) optimiser. Both the learning rate and weight decay were set to 0.001. For the spatio-temporal approach the batch size was set to 8 video sequences, while it was set to 64 images for the spatial approach.

### Experimental Setup

For the ablation studies, a stratified 10-fold cross-validation was performed using the first batch of data, such that the data was randomly split ten times into 20% validation set and 80% training set. Note that the splitting into training and validation sets was done on a patient basis. Furthermore, we evaluated these trained models also on the held-out test set.

As classification metrics, we evaluated the area under the receiver operation characteristic (AUROC, one-vs-one), balanced accuracy, frequency-weighted F1-score, weighted precision, and weighted recall, as commonly used metrics. The multi-view AUROC was computed from the output probabilities of the most confident model selected by the majority voting. Results were averaged over the folds, and the mean and standard deviation were reported on patient level.

### Ablation Studies for Spatial Approach

For performing various ablation studies, we simplified the problem setting to a binary classification. We then discriminated between no PH (65% of the data) and PH, combining mild, moderate, and severe cases, in line with previous work (Zhang et al., 2018). Even though assessing the severity of PH is crucial for correct treatment, as the morbidity rate significantly increases for higher degrees of PH (Corris & Degano, 2014; Galiè et al., 2015), the clinicians might also be interested in simply discriminating between healthy and unhealthy patients as an initial screening procedure. In such a case, the data imbalance would be less significant. To understand the importance of different regularisation methods, and the effects of different frame extraction methods, ablation studies were performed on the simpler task of single-view binary PH detection, for the PSAX-P view, using a spatial-only approach.

#### Augmentation and Regularisation

We report in Table 2 the results of regularisation techniques when applied to the spatial PSAX-P model for PH detection, specifically the following: Augmentation (*aug*), weight decay of 0.001 (*wd*), and initialising the model with pre-trained weights (*pre-trained*). The weight decay value of 0.001 was set empirically. For this study, we keep the number of frames per ECHO fixed, extracting 10 random frames from each ECHO.

The results clearly demonstrate the significance of regularisation in improving the performance. We observed a clear improvement in using all three regularisation techniques compared to no regularisation. This finding is consistent with our expectation that overfitting is more likely to occur in small datasets, and regularisation can help mitigate this issue. We found that data augmentation had the most substantial positive impact on performance compared to other regularisation techniques. We apply all three regularisation techniques for subsequent experiments, both for spatial and spatio-temporal approach.

**Table 2:** Effects of different regularisation methods, i.e *pre-trained weights*, *augmentation* (*aug*), and *weight decay* (*wd*), on the PH detection with the spatial PSAX-P model, when training on 10 random frames per subject

	Regularisation	AUROC	F1-Score	Precision	Recall	Balanced Accuracy	Confidence
Random	Baseline	$0.76 \pm 0.07$	$0.72 \pm 0.04$	$0.77 \pm 0.05$	$0.72 \pm 0.05$	$0.72 \pm 0.05$	$0.85 \pm 0.02$
	wd	$0.79 \pm 0.09$	$0.73 \pm 0.11$	$0.80 \pm 0.04$	$0.73 \pm 0.10$	$0.74 \pm 0.07$	$0.85 \pm 0.03$
	aug	$0.91 \pm 0.04$	$0.89 \pm 0.05$	$0.91 \pm 0.04$	$0.89 \pm 0.05$	$0.89 \pm 0.05$	$0.85 \pm 0.02$
	aug, wd	$0.91 \pm 0.03$	$0.89 \pm 0.04$	$0.90 \pm 0.03$	$0.89 \pm 0.04$	$0.89 \pm 0.04$	$0.85 \pm 0.02$
Pre-trained	Baseline	$0.86 \pm 0.04$	$0.82 \pm 0.05$	$0.84 \pm 0.05$	$0.82 \pm 0.05$	$0.81 \pm 0.05$	$0.87 \pm 0.02$
	wd	$0.86 \pm 0.07$	$0.83 \pm 0.07$	$0.84 \pm 0.06$	$0.83 \pm 0.07$	$0.82 \pm 0.06$	$0.87 \pm 0.02$
	aug	$0.92 \pm 0.05$	$0.90 \pm 0.04$	$0.92 \pm 0.03$	$0.90 \pm 0.04$	$0.91 \pm 0.04$	$0.86 \pm 0.03$
	**aug, wd**	${{\textbf {0.93}}} \pm {{\textbf {0.04}}}$	${{\textbf {0.91}}} \pm {{\textbf {0.03}}}$	${{\textbf {0.92}}} \pm {{\textbf {0.03}}}$	${{\textbf {0.91}}} \pm {{\textbf {0.03}}}$	${{\textbf {0.92}}} \pm {{\textbf {0.03}}}$	${{\textbf {0.87}}} \pm {{\textbf {0.03}}}$

The best results are highlighted in bold

#### Extraction of Frames

This study investigates the impact of using different methods to extract ECHO frames. Specifically, we compare the performance of a spatial single-view PH detection model, trained on around 10 frames from the PSAX-P view, corresponding to maximum-expansion frames (*Max 90*_th_), minimum-expansion frames (*Min 90*_th_), and random frames (*Rand*-10). As a baseline, we also show the results from training on *all* frames.

Our experimental results, reported in Table 3, indicate that selecting the frames corresponding to the minimum-expansion of the heart is more effective than selecting those corresponding to the maximum-expansion. This suggests that the minimum-expansion frames contain more informative features for detecting PH.

**Table 3:** Results of PH detection with the spatial PSAX-P model, when varying the method of extracting frames, i.e. maximum-expansion (*Max*), minimum-expansion (*Min*) or random (*Rand*). 90_th_ percentile correspond on average to 10–12 frames per ECHO

Frame Extraction	AUROC	F1-Score	Precision	Recall	Balanced Accuracy	Confidence
Max $90_{\textrm{th}}$	$0.87 \pm 0.04$	$0.87 \pm 0.03$	$0.88 \pm 0.03$	$0.87 \pm 0.04$	$0.86 \pm 0.03$	$0.89 \pm 0.01$
**Min** $\textbf{90}_{{\textbf {th}}}$	${\textbf {0.95}}\pm {\textbf {0.03}}$	${\textbf {0.92}}\pm {\textbf {0.04}}$	${\textbf {0.93}}\pm {\textbf {0.04}}$	${\textbf {0.92}}\pm {\textbf {0.04}}$	${\textbf {0.92}}\pm {\textbf {0.04}}$	${\textbf {0.88}}\pm {\textbf {0.02}}$
Rand 10	$0.93 \pm 0.04$	$0.91 \pm 0.03$	$0.92 \pm 0.03$	$0.91 \pm 0.03$	$0.92 \pm 0.03$	$0.87 \pm 0.03$
All	$0.94 \pm 0.03$	$0.91 \pm 0.03$	$0.92 \pm 0.03$	$0.91 \pm 0.03$	$0.90 \pm 0.04$	$0.86 \pm 0.02$

The best results are highlighted in bold

This finding is consistent with previous research. It shows that PH-related abnormalities visible from the PSAX-P view in newborns, such as changes in the shape of the IVS and LV, are most prominent during systole, i.e. when the heart is at its minimum-expansion (EL-Khuffash, 2014). Although PH is also associated with the reversed volume of the ventricles during diastole, the systolic changes in LV and IVS morphology appear to be more discriminative features for the spatial model. This is in line with ground-truth annotations being primarily based on IVS morphology and is further supported by our explainability analysis in the following sections (Sect. 4.5.1).

While best results are achieved using frames corresponding to the minimum expansion, the improvement compared to using randomly selected frames is negligible, with the balanced accuracy being the same and other metrics only slightly improved. We, thus, choose to train our model on random frames for future experiments. Using random frames aligns with previous work, creating a fair comparison and is more generalizable across different views.

An interesting observation is that training on random frames yields better results than training on maximum-expansion frames. This may be because by introducing randomness into the training process, the model has the opportunity to identify and incorporate a broader range of features that might not be immediately apparent to human observers. The maximum-expansion frames might not be discriminative enough in isolation. Finally, training on all frames does not give improvements over selecting only 10 frames.

### Ablation Studies for Spatio-Temporal Approach

We performed ablation studies to understand the importance of different 3D-CNN Architectures for extracting spatio-temporal features and the effects of different ECHO sequence lengths. The ablation studies were performed on the binary PH detection task using the PSAX-P view.

#### Different 3D-CNN Architectures

We evaluated different spatio-temporal architectures, such as ResNet3D (Hara et al., 2017), R(2+1)D (Tran et al., 2018) and SlowFast (Feichtenhofer et al., 2019). Table 4 provides an empirical evaluation for binary PH detection using PSAX-P view. The 18-layer ResNet3D (same results as in Table 6(b)) shows superior performance compared to the other two architectures, with SlowFast being slightly better than R(2+1)D. Although the 50-layer SlowFast network has shown superior performances on various video classification tasks (Feichtenhofer et al., 2019), it seems to lead to overfitting in our dataset. Furthermore, factoring the 3D convolutional filters into separate spatial and temporal components, as in R(2+1)D, does not improve accuracy in our case.

**Table 4:** Results from different spatio-temporal architectures for binary PH detection using PSAX-P view

Architecture	AUROC	F1-Score	Precision	Recall	Balanced Accuracy	Confidence
R(2+1)D - 18 layers	$0.90 \pm 0.06$	$0.90 \pm 0.03$	$0.91 \pm 0.03$	$0.90 \pm 0.03$	$0.90 \pm 0.05$	$0.91 \pm 0.03$
SlowFast - 50 layers	$0.93 \pm 0.04$	$0.90 \pm 0.04$	$0.91 \pm 0.04$	$0.90 \pm 0.04$	$0.90 \pm 0.05$	$0.90 \pm 0.03$
**ResNet3D - 18 layers**	${{\textbf {0.95}}} \pm {{\textbf {0.04}}}$	${{\textbf {0.92}}} \pm {{\textbf {0.03}}}$	${{\textbf {0.93}}} \pm {{\textbf {0.03}}}$	${{\textbf {0.92}}} \pm {{\textbf {0.03}}}$	${{\textbf {0.94}}} \pm {{\textbf {0.03}}}$	${{\textbf {0.91}}} \pm {{\textbf {0.01}}}$

The best results for each task have been highlighted in bold

#### Sequence Length and Sampling Interval

Given an ECHO sequence length *k* and sampling interval *s*, the effective sequence length *l* is defined as $l = k * s$, and it determines the number of frames the given sequence covers. The effective length can, in theory, be set to any number between 1 and *max* frames, where *max* is the length of the full ECHO, on average ${\textit{max}}=122$ frames. However, a sequence covering a single frame does not utilize the temporal information and using an entire ECHO as a sequence leads to slow training and provides fewer training samples.

We hypothesize a sequence covering at least a full heartbeat (i.e. 10–12 frames) will be necessary for the best performance. However, the ideal effective length, sequence length, and sampling interval are determined with an ablation study. Table 5 reports the effects of varying the effective length (*l*) of the input sequences from min 8 to max 24 for sampling intervals $s=1$ and $s=2$ when training a ResNet3D-18 model on the PSAX-P view, for the task of binary PH detection. These settings correspond to sequence lengths (*k*) in the range of 4 to 24.

**Table 5:** Results of PH detection with the ResNet3D PSAX-P model when varying the effective length (*l*), sequence-length (*k*) and sampling interval (*s*) of the input sequences

*k*, *s* (*l*)	AUROC	F1-Score	Precision	Recall	Balanced Accuracy	Confidence
8, 1 (8)	$0.92 \pm 0.03$	$0.90 \pm 0.04$	$0.92 \pm 0.02$	$0.90 \pm 0.04$	$0.91 \pm 0.03$	$0.90 \pm 0.02$
**12, 1 (12)**	${{\textbf {0.95}}} \pm {{\textbf {0.04}}}$	${{\textbf {0.92}}} \pm {{\textbf {0.03}}}$	${{\textbf {0.93}}} \pm {{\textbf {0.03}}}$	${{\textbf {0.92}}} \pm {{\textbf {0.03}}}$	${{\textbf {0.94}}} \pm {{\textbf {0.03}}}$	${{\textbf {0.90}}} \pm {{\textbf {0.03}}}$
16, 1 (16)	$0.92 \pm 0.04$	$0.90 \pm 0.04$	$0.91 \pm 0.03$	$0.89 \pm 0.04$	$0.91 \pm 0.04$	$0.92 \pm 0.02$
24, 1 (24)	$0.92 \pm 0.04$	$0.88 \pm 0.03$	$0.90 \pm 0.02$	$0.87 \pm 0.03$	$0.89 \pm 0.03$	$0.93 \pm 0.01$
4, 2 (8)	$0.92 \pm 0.04$	$0.89 \pm 0.05$	$0.91 \pm 0.03$	$0.89 \pm 0.05$	$0.90 \pm 0.04$	$0.89 \pm 0.02$
6, 12 (12)	$0.92 \pm 0.03$	$0.91 \pm 0.03$	$0.92 \pm 0.02$	$0.91 \pm 0.03$	$0.92 \pm 0.03$	$0.90 \pm 0.01$
8, 2 (16)	$0.92 \pm 0.04$	$0.91 \pm 0.03$	$0.92 \pm 0.03$	$0.91 \pm 0.03$	$0.91 \pm 0.03$	$0.92 \pm 0.02$
12, 2 (24)	$0.94 \pm 0.03$	$0.92 \pm 0.03$	$0.92 \pm 0.03$	$0.92 \pm 0.03$	$0.92 \pm 0.04$	$0.93 \pm 0.01$

The best results have been highlighted in bold

The best results are achieved when training on input sequences of length 12, where every consecutive frame is selected (i.e. $l=12$, $k=12$, $s=1$). Recall that a single heartbeat covers, on average, 10 frames, so when the input sequences cover 12 frames, they contain at least one heartbeat on average. The second-best performance is also achieved with sequences of length $k=12$, but by sampling every other frame, such that the effective length is 24 frames, $l=24$, $k=12$, $s=2$. In general we can see that training on sequences with the same effective length but varying the sampling rate yields similar results.

### Results of Proposed Approach and Discussions

We hereby provide an empirical assessment of the proposed approaches for PH severity prediction on the dataset described in Sect. 3.1. Note that, we used the ablation study results from the previous section to select the best model and parameters. We report the quantitative performance from each of the three major views (A4C, PLAX, PSAX-P) (see Fig. 3a) and two minor views (PSAX-S, PSAX-A). We also report the results from the multi-view approach (see Fig. 3b) obtained through majority voting using different combinations of views. In particular, we combined the 3 major views (MV-3) and all views for a total of 5 different views (MV-All).

**Table 6:** Results from both spatio-temporal (a, b) and spatial (c, d) approaches for PH severity prediction (a,c) and binary PH detection (b,d) using *10-fold cross-validation*. *MV-3* refers to majority voting of A4C, PLAX, and, PSAX-P views

	View	AUROC	F1-Score	Precision	Recall	Balanced accuracy	Confidence
(a) Spatio-temp. Severity prediction	A4C	$0.77 \pm 0.03$	$0.72 \pm 0.05$	$0.75 \pm 0.05$	$0.72 \pm 0.05$	$0.65 \pm 0.06$	$0.88 \pm 0.02$
	PLAX	$0.85 \pm 0.04$	$0.78 \pm 0.05$	$0.82 \pm 0.06$	$0.79 \pm 0.06$	$0.72 \pm 0.05$	$0.89 \pm 0.03$
	PSAX-P	$0.85 \pm 0.04$	$0.81 \pm 0.05$	$0.83 \pm 0.06$	$0.82 \pm 0.04$	$0.73 \pm 0.06$	$0.90 \pm 0.03$
	PSAX-S	$0.73 \pm 0.07$	$0.68 \pm 0.08$	$0.69 \pm 0.09$	$0.69 \pm 0.08$	$0.62 \pm 0.07$	$0.85 \pm 0.04$
	PSAX-A	$0.77 \pm 0.07$	$0.74 \pm 0.06$	$0.77 \pm 0.04$	$0.74 \pm 0.06$	$0.67 \pm 0.06$	$0.84 \pm 0.04$
	MV-3	$0.84 \pm 0.08$	$0.83 \pm 0.05$	$0.86 \pm 0.04$	$0.83 \pm 0.05$	$0.76 \pm 0.07$	$0.91 \pm 0.02$
	**MV-All**	${{\textbf {0.86}}} \pm {{\textbf {0.09}}}$	${{\textbf {0.84}}} \pm {{\textbf {0.06}}}$	${{\textbf {0.86}}} \pm {{\textbf {0.05}}}$	${{\textbf {0.85}}} \pm {{\textbf {0.05}}}$	${{\textbf {0.78}}} \pm {{\textbf {0.07}}}$	${{\textbf {0.90}}} \pm {{\textbf {0.02}}}$
(b) Spatio-temp. Binary detection	A4C	$0.83 \pm 0.05$	$0.81 \pm 0.04$	$0.84 \pm 0.03$	$0.81 \pm 0.04$	$0.81 \pm 0.04$	$0.91 \pm 0.03$
	PLAX	$0.90 \pm 0.07$	$0.86 \pm 0.09$	$0.88 \pm 0.07$	$0.86 \pm 0.09$	$0.86 \pm 0.08$	$0.91 \pm 0.02$
	**PSAX-P**	${{\textbf {0.95}}} \pm {{\textbf {0.04}}}$	${{\textbf {0.92}}} \pm {{\textbf {0.03}}}$	${{\textbf {0.93}}} \pm {{\textbf {0.03}}}$	${{\textbf {0.92}}} \pm {{\textbf {0.03}}}$	${{\textbf {0.94}}} \pm {{\textbf {0.03}}}$	${{\textbf {0.90}}} \pm {{\textbf {0.03}}}$
	PSAX-S	$0.79 \pm 0.04$	$0.81 \pm 0.03$	$0.82 \pm 0.04$	$0.81 \pm 0.03$	$0.80 \pm 0.04$	$0.90 \pm 0.02$
	PSAX-A	$0.88 \pm 0.05$	$0.87 \pm 0.03$	$0.88 \pm 0.03$	$0.87 \pm 0.03$	$0.87 \pm 0.04$	$0.89 \pm 0.03$
	MV-3	$0.90 \pm 0.03$	$0.87 \pm 0.04$	$0.88 \pm 0.03$	$0.87 \pm 0.04$	$0.87 \pm 0.04$	$0.92 \pm 0.01$
	MV-All	$0.90 \pm 0.03$	$0.89 \pm 0.02$	$0.90 \pm 0.02$	$0.89 \pm 0.02$	$0.89 \pm 0.02$	$0.91 \pm 0.01$
(c) Spatial Severity prediction	A4C	$0.79 \pm 0.04$	$0.75 \pm 0.05$	$0.77 \pm 0.04$	$0.75 \pm 0.06$	$0.67 \pm 0.06$	$0.84 \pm 0.03$
	PLAX	$0.84 \pm 0.04$	$0.76 \pm 0.04$	$0.78 \pm 0.05$	$0.77 \pm 0.04$	$0.70 \pm 0.05$	$0.85 \pm 0.03$
	PSAX-P	$0.83 \pm 0.03$	$0.81 \pm 0.02$	$0.82 \pm 0.03$	$0.81 \pm 0.03$	$0.74 \pm 0.03$	$0.83 \pm 0.03$
	PSAX-S	$0.74 \pm 0.07$	$0.68 \pm 0.06$	$0.70 \pm 0.07$	$0.70 \pm 0.08$	$0.62 \pm 0.06$	$0.83 \pm 0.03$
	PSAX-A	$0.80 \pm 0.03$	$0.75 \pm 0.04$	$0.76 \pm 0.04$	$0.76 \pm 0.05$	$0.66 \pm 0.04$	$0.84 \pm 0.03$
	MV-3	$0.84 \pm 0.03$	$0.81 \pm 0.03$	$0.83 \pm 0.04$	$0.82 \pm 0.03$	$0.74 \pm 0.06$	$0.85 \pm 0.02$
	MV-All	$0.84 \pm 0.03$	$0.82 \pm 0.03$	$0.83 \pm 0.04$	$0.83 \pm 0.03$	$0.73 \pm 0.04$	$0.85 \pm 0.01$
(d) Spatial Binary detection	$\text {A4C}^{*}$	$0.87 \pm 0.04$	$0.83 \pm 0.04$	$0.85 \pm 0.03$	$0.83 \pm 0.04$	$0.83 \pm 0.03$	$0.87 \pm 0.03$
	PLAX	$0.92 \pm 0.05$	$0.88 \pm 0.04$	$0.89 \pm 0.04$	$0.88 \pm 0.04$	$0.88 \pm 0.04$	$0.89 \pm 0.02$
	PSAX-P	$0.93 \pm 0.04$	$0.91 \pm 0.03$	$0.92 \pm 0.03$	$0.91 \pm 0.03$	$0.92 \pm 0.03$	$0.87 \pm 0.03$
	PSAX-S	$0.83 \pm 0.03$	$0.81 \pm 0.03$	$0.83 \pm 0.02$	$0.81 \pm 0.03$	$0.81 \pm 0.03$	$0.86 \pm 0.04$
	PSAX-A	$0.86 \pm 0.04$	$0.85 \pm 0.03$	$0.85 \pm 0.03$	$0.85 \pm 0.03$	$0.84 \pm 0.03$	$0.87 \pm 0.02$
	MV-3	$0.91 \pm 0.02$	$0.88 \pm 0.02$	$0.88 \pm 0.02$	$0.88 \pm 0.02$	$0.88 \pm 0.02$	$0.88 \pm 0.01$
	MV-All	$0.92 \pm 0.02$	$0.90 \pm 0.02$	$0.91 \pm 0.01$	$0.90 \pm 0.02$	$0.90 \pm 0.01$	$0.87 \pm 0.02$

*MV-All* refers to majority voting of all five views. The best results for each task have been highlighted in bold

We report PH severity prediction performance in Table 6(a). Among the single-view methods, the parasternal short-axis view at the level of papillary muscles (PSAX-P) shows the best performance in PH severity prediction. It achieves an F1-score of 0.81 and Balanced Accuracy of 0.73, followed by the parasternal long-axis view (PLAX). Although the apical four-chamber view (A4C) is one of the most commonly used views for cardiovascular disease diagnosis, our evaluation shows that it is not as discriminative as PSAX-P and PLAX, yielding an F1-score of 0.72 and Balanced Accuracy of 0.65, which are clearly lower than the other two views. This is also in line with the neonatal echocardiography teaching manual (EL-Khuffash, 2014), where it is stated that subjective assessment of PH from the A4C view in a 2D ECHO is usually only possible for moderate to severe PH cases, and quantitative evaluation is difficult.

The PH severity prediction problem is challenging, not only due to the complex task at hand but also because of the data imbalance. In this case, the robustness and accuracy can be increased by utilising more views. By combining results from the PSAX-P, PLAX, and A4C views using majority voting, the F1-score increased to 0.83, while the Balanced Accuracy to 0.76. This improvement surpasses the performance of any of the single views. When the other two short-axis views are included, we get an F1-score of 0.84 and a Balanced Accuracy of 0.78. The majority voting is not only helpful to enhance the performance, but it is also useful in case a single view has an unsatisfactory quality for a given subject, a common scenario in many real-world applications.

Moreover, we performed an additional ablation, where we simplified the problem setting to a binary classification, as described in Sect. 4.3. We report the binary PH detection results in Table 6(b). PSAX-P view is still the most discriminative one with an F1-score of 0.92 and Balanced Accuracy of 0.94. Given the substantial prediction accuracy of the PSAX-P view alone for binary PH detection, including more views to the aggregated model does not result in increased performances. This is due to the larger number of weaker models and the high performance of the PSAX-P single view. The voting strategy is then driven by weaker models rather than the single best performing PSAX-P view.

In addition, we also explored an alternative end-to-end approach by merging the views in the embedding space. This was considered as an alternative method for combining views instead of majority voting. However, this approach did not yield improved results compared to the majority voting. Furthermore, we investigated confidence-weighted majority voting. While the average confidence is higher for correct predictions, there were some cases where the models confidently made incorrect predictions. Thus, we decided against using voting weighted by confidence.

The existing method (Zhang et al., 2018) for binary PH detection in adults does not exploit the spatio-temporal patterns. Thus, as a comparison, we also evaluated the spatial-only approach. The results are reported in Table 6(c) for severity prediction and in Table 6(d) for binary PH detection. We achieve similar results as the state-of-the-art method using A4C view as in Zhang et al. (2018), with an AUROC of 0.87 compared to 0.85 from Zhang et al. (2018), when evaluating a similar task in adults. In terms of severity prediction using a spatial only approach, from Table 6(c), MV-All demonstrates (slightly) higher performance over PSAX-P. Furthermore, when comparing the spatio-temporal method in Table 6(a) with the spatial method in Table 6(c), the utilization of the spatio-temporal method with MV-All helps enhancing the overall performance, improving the Balanced Accuracy from 0.73 to 0.78. Regarding binary PH detection, the comparison between the spatio-temporal method in Table 6(b) and the spatial method in Table 6(d) indicates that employing a single and highly predictive PSAX-P view yields the best outcomes for both methods. As a future work, we aim to improve the aggregation strategy from multi views for binary detection.

We evaluated our models on the held-out test set, and these results are presented in Table 7. In Table 7(a), our MV-All method achieved an F1 score of 0.63 for severity prediction on the test set, compared to 0.84 in the validation set. Furthermore, the PSAX-A view is the most predictive view for the severity prediction task on the test set, closely followed by PLAX. Regarding the binary PH detection, shown in Table 7(b), our MV-All method achieved an F1 score of 0.78, compared to the best accuracy using PSAX-P of 0.92 in the validation set. For severity prediction, shown in Table 7(a) and (c), employing the spatio-temporal approach resulted in better scores. In contrast, for binary PH detection, the spatial approach yielded higher scores with both single- and multi-view methods, as shown in Table 7(b) and (d). Overall, the models demonstrated high accuracy for the binary PH detection task on the unseen test set. However, as severity prediction presents a more challenging task, these models might benefit from additional data to better differentiate between varying levels of PH in future iterations, potentially enhancing accuracy.

**Table 7:** Results from both spatio-temporal (a, b) and spatial (c, d) approaches for PH severity prediction (a, c) and binary PH detection (b, d) on *held-out test data*

	View	AUROC	F1-Score	Precision	Recall	Balanced Accuracy	Confidence
(a) Spatio-temp. Severity prediction	A4C	$0.72 \pm 0.06$	$0.58 \pm 0.08$	$0.59 \pm 0.06$	$0.64 \pm 0.09$	$0.45 \pm 0.07$	$0.88 \pm 0.06$
	PLAX	$0.72 \pm 0.07$	$0.65 \pm 0.03$	$0.65 \pm 0.05$	$0.69 \pm 0.04$	$0.44 \pm 0.04$	$0.90 \pm 0.03$
	PSAX-P	$0.76 \pm 0.06$	$0.60 \pm 0.07$	$0.61 \pm 0.10$	$0.66 \pm 0.06$	$0.48 \pm 0.08$	$0.88 \pm 0.02$
	PSAX-S	$0.73 \pm 0.08$	$0.58 \pm 0.07$	$0.59 \pm 0.07$	$0.60 \pm 0.08$	$0.48 \pm 0.07$	$0.81 \pm 0.04$
	PSAX-A	$0.79 \pm 0.04$	$0.66 \pm 0.05$	$0.67 \pm 0.07$	$0.69 \pm 0.05$	$0.50 \pm 0.07$	$0.88 \pm 0.03$
	MV-3	$0.73 \pm 0.06$	$0.61 \pm 0.02$	$0.62 \pm 0.03$	$0.68 \pm 0.03$	$0.46 \pm 0.03$	$0.91 \pm 0.03$
	MV-All	$0.74 \pm 0.06$	$0.63 \pm 0.03$	$0.63 \pm 0.02$	$0.70 \pm 0.02$	$0.48 \pm 0.04$	$0.89 \pm 0.02$
(b) Spatio-temp. Binary detection	A4C	$0.83 \pm 0.04$	$0.71 \pm 0.05$	$0.74 \pm 0.05$	$0.73 \pm 0.04$	$0.67 \pm 0.05$	$0.89 \pm 0.02$
	PLAX	$0.82 \pm 0.08$	$0.68 \pm 0.06$	$0.77 \pm 0.05$	$0.72 \pm 0.05$	$0.63 \pm 0.07$	$0.92 \pm 0.04$
	PSAX-P	$0.83 \pm 0.05$	$0.72 \pm 0.06$	$0.77 \pm 0.04$	$0.74 \pm 0.05$	$0.71 \pm 0.06$	$0.90 \pm 0.04$
	PSAX-S	$0.84 \pm 0.07$	$0.75 \pm 0.06$	$0.77 \pm 0.06$	$0.75 \pm 0.06$	$0.75 \pm 0.06$	$0.84 \pm 0.03$
	PSAX-A	$0.85 \pm 0.05$	$0.78 \pm 0.04$	$0.81 \pm 0.05$	$0.79 \pm 0.04$	$0.73 \pm 0.05$	$0.92 \pm 0.04$
	MV-3	$0.83 \pm 0.07$	$0.73 \pm 0.07$	$0.81 \pm 0.03$	$0.76 \pm 0.05$	$0.70 \pm 0.06$	$0.92 \pm 0.01$
	MV-All	$0.84 \pm 0.07$	$0.78 \pm 0.04$	$0.84 \pm 0.03$	$0.80 \pm 0.03$	$0.75 \pm 0.04$	$0.91 \pm 0.01$
(c) Spatial Severity prediction	A4C	$0.70 \pm 0.10$	$0.54 \pm 0.04$	$0.52 \pm 0.07$	$0.62 \pm 0.04$	$0.39 \pm 0.04$	$0.89 \pm 0.04$
	PLAX	$0.76 \pm 0.06$	$0.64 \pm 0.05$	$0.63 \pm 0.10$	$0.71 \pm 0.04$	$0.43 \pm 0.08$	$0.93 \pm 0.03$
	PSAX-P	$0.79 \pm 0.02$	$0.60 \pm 0.05$	$0.64 \pm 0.09$	$0.66 \pm 0.03$	$0.47 \pm 0.05$	$0.85 \pm 0.04$
	PSAX-S	$0.73 \pm 0.07$	$0.60 \pm 0.07$	$0.59 \pm 0.08$	$0.64 \pm 0.06$	$0.46 \pm 0.07$	$0.85 \pm 0.03$
	PSAX-A	$0.84 \pm 0.04$	$0.66 \pm 0.05$	$0.68 \pm 0.06$	$0.71 \pm 0.04$	$0.49 \pm 0.09$	$0.89 \pm 0.03$
	MV-3	$0.73 \pm 0.06$	$0.58 \pm 0.02$	$0.58 \pm 0.05$	$0.66 \pm 0.02$	$0.42 \pm 0.03$	$0.90 \pm 0.02$
	MV-All	$0.72 \pm 0.05$	$0.58 \pm 0.03$	$0.60 \pm 0.07$	$0.66 \pm 0.02$	$0.42 \pm 0.03$	$0.90 \pm 0.02$
(d) Spatial Binary detection	A4C	$0.81 \pm 0.06$	$0.71 \pm 0.06$	$0.76 \pm 0.05$	$0.74 \pm 0.04$	$0.68 \pm 0.07$	$0.90 \pm 0.03$
	PLAX	$0.79 \pm 0.07$	$0.73 \pm 0.05$	$0.80 \pm 0.06$	$0.77 \pm 0.04$	$0.66 \pm 0.05$	$0.95 \pm 0.02$
	PSAX-P	$0.90 \pm 0.04$	$0.79 \pm 0.04$	$0.82 \pm 0.05$	$0.80 \pm 0.04$	$0.77 \pm 0.04$	$0.89 \pm 0.02$
	PSAX-S	$0.90 \pm 0.05$	$0.80 \pm 0.04$	$0.82 \pm 0.05$	$0.80 \pm 0.04$	$0.78 \pm 0.04$	$0.86 \pm 0.03$
	PSAX-A	$0.89 \pm 0.05$	$0.83 \pm 0.04$	$0.85 \pm 0.04$	$0.84 \pm 0.04$	$0.80 \pm 0.05$	$0.90 \pm 0.02$
	MV-3	$0.86 \pm 0.09$	$0.78 \pm 0.05$	$0.84 \pm 0.03$	$0.80 \pm 0.04$	$0.75 \pm 0.05$	$0.92 \pm 0.01$
	MV-All	$0.86 \pm 0.08$	$0.80 \pm 0.04$	$0.86 \pm 0.01$	$0.82 \pm 0.03$	$0.77 \pm 0.04$	$0.91 \pm 0.01$

*MV-3* refers to majority voting of A4C, PLAX, and, PSAX-P views. *MV-All* refers to majority voting of all five views

#### Explainability

**Fig. 4:**
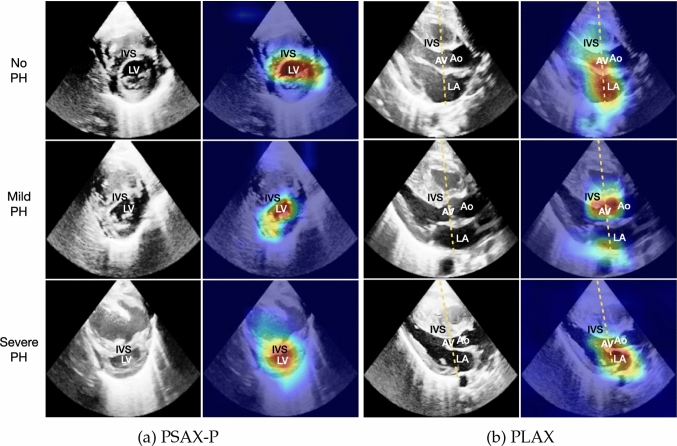
ECHO frames of subjects with no, mild and severe PH (left), as well as the IP-PHN saliency maps (right), for **a** the PSAX-P view, and **b** the PLAX view. The yellow line shows how the M-mode for the LA:Ao measurement is extracted. The highlighted pixels feature crucial cardiac structures

To increase the clinical usability, our method contains a post-hoc analysis of the single-view spatio-temporal convolutions. For each ECHO view, we highlight the pixels that are the most relevant for assessing PH severity. In Fig. 4, we show the original ECHO frames with different levels of PH (left column) combined with saliency maps using Grad-CAM (right column) corresponding to the significant views, in Fig. 4a PSAX-P and in Fig. 4b PLAX views.

According to the neonatal echocardiography teaching manual (EL-Khuffash, 2014), for the PSAX-P view, PH results in change in the IVS morphology and LV shape, which stems from the change in RV pressure. In mild to moderate PH, the IVS becomes flat during systole. In moderate to severe PH, the septum bows into the LV, such that the LV becomes D-shaped or crescentic. We show in Fig. 4a that our PSAX-P severity prediction model evaluates the change in the shape of LV, which is the result of an enlarged RV. Thus, our model focuses on the same clinically relevant features as are recommended for diagnosis.

Subjective evaluation of the IVS morphology is also possible from the PLAX view (EL-Khuffash, 2014). Furthermore, quantitative assessments are frequently performed on this view. These assessments include measurements evaluating left atrial filling like left atrial-to-aortic root diameter ratio (LA:Ao) by extracting the M-mode as demonstrated with the yellow line in Fig. 4b. When exploring the saliency map of the PLAX severity model, we see in Fig. 4b that the model focuses on the area around the LA, AV and Ao, and IVS. This suggests that the model is able to consider both the relevant quantitative features and the subjective ones.

**Fig. 5:**
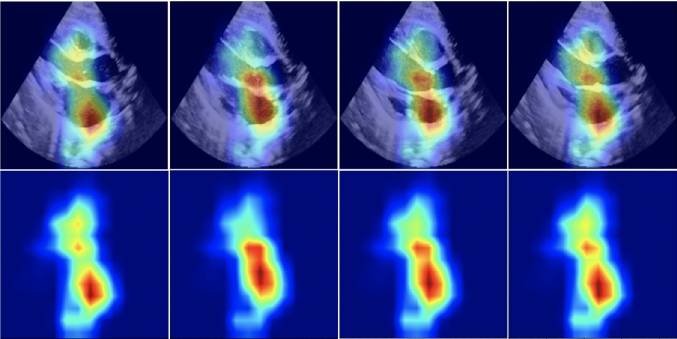
Spatio-temporal Grad-CAM saliency maps (bottom) imposed on the original frames (top) for frames corresponding to systole, mid, diastole, mid in a PLAX ECHO

Note that, for simplicity, we show the visualisation results of a single frame per patient in Fig. 4. In a clinical setting, the visualizations can be viewed as a video containing spatio-temporal explanation. In Fig. 5, we show more examples of how the focus changes along the frames of a sequence.

We would like to stress that, although we only plotted a few random individuals, we analyzed saliency maps of the entire population to draw meaningful conclusions. Publicly available provided code can be used to test such findings.

## Conclusion

In this study, we developed an automated and streamlined approach to assist clinicians in assessing pulmonary hypertension (PH) in newborns using echocardiography (ECHO), which remains a challenge for cardiologists (Dasgupta et al., 2021; Fisher et al., 2009).

To estimate PH severity, we experimented with spatial-only as well as spatio-temporal approaches from one or multiple views. The optimal performance was achieved using a spatio-temporal convolutional model on multiple views and using the majority voting of those as the final prediction for the validation set. For the held-out test set, the models can benefit from more data to improve the accuracy. Our method has the potential to significantly improve the accuracy, reliability, and consistency of PH estimation in newborns, thereby reducing the number of missed or delayed diagnoses of severe PH. As the severity of PH determines the urgency of treatment (Corris & Degano, 2014; Galiè et al., 2015), this, in turn, could improve the prognosis of PH patients by allowing for earlier treatment.

Collecting ECHOs from multiple views requires specialized expertise, and integrating these into the model can be time-intensive. However, our approach presents a standardized and more objective method for detecting PH and assessing its severity. This method complements and potentially enhances the expertise-driven assessments. Furthermore, the models can be further refined by incorporating multi-annotator labels through re-training, aiming to further reduce subjectivity. Moreover, our approach may assist less trained specialists and reduce the workload of highly trained experts. Additionally, by highlighting the input features that are crucial for PH assessment, our proposed method provides understandable explanations for clinicians, making the system accountable.

It is important to note that this study focused on analyzing the shape change and motion of the ventricles and septum using 2D echos in standard planes and was conducted retrospectively. However, integrating other modalities, such as Doppler echocardiography or electrocardiograms (ECGs), could extend the method to detect abnormalities in cardiac functions. Furthermore, the proposed method is not limited to newborns and could be applied to ECHOs from the adult population, with re-training required. Finally, due to the multi-view setup, the pipeline could be adapted to other types of disease predictions in the medical context, where clinicians benefit from different views of the heart in their clinical routine.

## Data Availability

The dataset analysed during the current study is available from the corresponding author on reasonable request.
